# The expression and role of tenascin C in abdominal aortic aneurysm formation and progression

**DOI:** 10.1093/icvts/ivac018

**Published:** 2022-02-08

**Authors:** Felix Nagel, Anne-Kristin Schaefer, Inês Fonseca Gonçalves, Eylem Acar, Andre Oszwald, Philipp Kaiser, Renate Kain, Karola Trescher, Wolf H Eilenberg, Christine Brostjan, David Santer, Attila Kiss, Bruno K Podesser

**Affiliations:** 1 Ludwig Boltzmann Institute for Cardiovascular Research, Center for Biomedical Research, Medical University of Vienna, Vienna, Austria; 2 Department of Cardiac Surgery, University Hospital St. Pölten, Karl Landsteiner University, St. Pölten, Austria; 3 Department of Pathology, Medical University of Vienna, Vienna, Austria; 4 Department of Vascular Surgery, Medical University of Vienna, Vienna, Austria; 5 Department of Cardiac Surgery, University Hospital Basel, Basel, Switzerland

**Keywords:** Abdominal aortic aneurysm, Tenascin C, Serum marker, Extracellular matrix

## Abstract

**OBJECTIVES:**

Up-regulation of tenascin C (TNC), a matricellular protein, produced mainly by vascular smooth muscle cells (VSMC), is associated with the progression and dilation of abdominal aortic aneurysms (AAA). The aims of this study were (i) to evaluate whether serum levels of TNC in patients with AAA patients correlate with aortic diameter and (ii) to clarify the role of TNC in formation and progression of AAA in a murine model.

**METHODS:**

In 15 patients with AAA serum levels of TNC were measured and correlated with aortic diameters. Moreover, in a murine calcium chloride AAA model, the impact of TNC deficiency on AAA diameter was evaluated. Finally, human VSMC were incubated with TNC to clarify its regulating potential.

**RESULTS:**

In the clinical cohort, there was a trend of correlation between serum TNC levels and AAA diameter (*P* = 0.055). TNC knock out mice with AAA showed significantly lower diameter ratios compared to the wild-type group (WT) 3 weeks (*P* < 0.05) and 10 weeks (*P* < 0.05) after AAA induction. Immunohistochemistry revealed increased TNC expression in aortic tissue from WT with AAA as compared sham-operated mice. Furthermore, WT with AAA showed a more disrupted Elastin structure than TNC knock out mice 10 weeks after AAA induction. In human aortic VSMC, TNC incubation induced expression of remodelling associated proteins.

**CONCLUSIONS:**

TNC might play a causative role in the formation, dilation and progression of AAA. Our results indicate that TNC might be a biomarker as well as a potential therapeutic target in the treatment of AAA.

**Figure:**
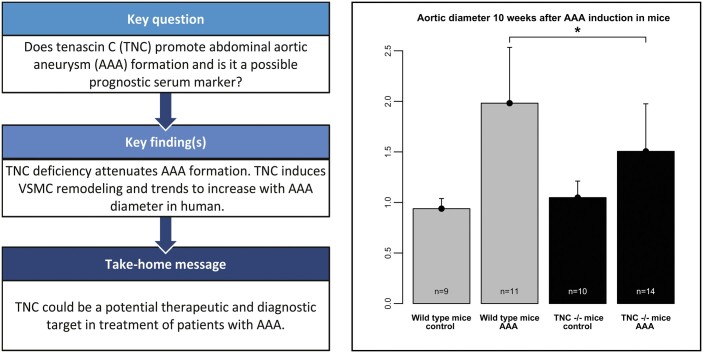

## INTRODUCTION

Abdominal aortic aneurysms (AAA) are defined as dilations of the abdominal aorta with more than 30 mm diameter in men and are a risk factor for rupture [1]. Therefore, in asymptomatic AAA patients with no additional risk factors, an abdominal aorta diameter of more than 50–55 mm is recommended as indication for surgical or endovascular repair [1]. While management of risk factors e.g. arterial hypertension and smoking is standard of care, no causal medical treatment has yet proven to be an advantage in randomized placebo-controlled trials [1, 2].

Mechanistically, chronic inflammation is associated with the development of AAA leading to vascular smooth muscle cells (VSMC) proliferation and apoptosis as well as extracellular matrix dysregulation [2]. More recently, experimental studies demonstrated that the up-regulation of tenascin C (TNC), a matricellular glycoprotein, correlates with AAA progression [3, 4]. Moreover, our group showed that an elevated TNC expression in patients with acute Type A dissection of the ascending aorta, probably acts as a factor of destabilization [5]. Similarly, previous preclinical and clinical studies provided further evidence that pathophysiologic adverse remodelling has been improved in TNC deficient mice with pressure overload induced left ventricular hypertrophy or myocardial infarction [6, 7].

The present study aimed (i) to evaluate whether TNC serum levels correlate with aortic diameters in patients with AAA and (ii) to clarify the causative role of TNC on AAA formation in a murine TNC knockout model as well as human aortic VSMC culture.

## MATERIALS AND METHODS

### Patient cohort of abdominal aortic aneurysms

The study was approved by the institutional ethics committee at the Medical University of Vienna (1729/2014) and performed according to the Declaration of Helsinki. Due to the confounding effects on TNC expression, exclusion criteria were: active infections and carcinoma diagnosed during the preceding 5 years [8–10]. TNC levels in serum were measured by enzyme-linked immunosorbent assay (ab213831, Abcam, Cambridge, UK) according to the manufacturer’s recommendations. Syngo.via—CT vascular software (V. 5.1, Siemens Healthcare GmbH, Erlangen, Germany) was used for diameter measurements as well as three-dimensional reconstructions. The maximum aortic diameter in computed tomography angiography was measured perpendicularly to the aortic axis after multiplanar reformation.

### Murine abdominal aortic aneurysm model and organ preparation

The experimental protocol was approved by the Ethics Committee for Laboratory Animal Experiments at the Medical University of Vienna and the Austrian Ministry of Science Research and Economy (BMWFW-66.009/0278-II/3b/2012) as well as conforms with the Guide for the Care and Use of Laboratory Animals published by the US National Institutes of Health (NIH Publication No. 85-23, revised 1996).

In male, 9-week-old A/J wild type (WT) and A/J TNC knock out mice [A/J-TgH(Tnc), RBRC00007, RIKEN BioResource Center, Tsukuba, Ibaraki, Japan] AAA were induced by periaortic calcium chloride (CaCl_2_) application, as described by Chiou *et al.* [11–14]. Shortly, after subcutaneous administration of buprenorphine (0.1 mg/kg body weight) and sedation with 4% isoflurane, mice were intubated. Under continuous ventilation with 2% isoflurane, median laparotomy was performed. After preparation of the infrarenal aorta, its diameter was measured with the operating microscope, as described by Chiou *et al.* [14]. Subsequently, a 5 × 10 mm piece of cotton gauze soaked with 0.5 M CaCl_2_ (Amresco E506-100ML CaCl_2_ 1 M Sterile Solution, VWR International GmbH, Darmstadt, Germany; diluted 1:1 with 0.9% sodium chloride) was applied on the surface of the aorta for 15 min. The gauze was removed and the laparotomy was closed. After recovery of spontaneous breathing, mice were extubated. Piritramide (30 mg piritramide diluted in 10 ml Glucose 10% + 250 ml Water) in the drinking water was used for postoperative analgesia. Sham groups were treated identically, but sodium chloride (0.9%) was used instead of CaCl_2_ application.

Aortic diameter measurement and tissue harvesting were performed 3 and 10 weeks after AAA induction under deep sedoanalgesia with intraperitoneal administration of ketamine (100 mg/kg body weight) and xylazine (5 mg/kg body weight). Again, the infrarenal aorta was prepared and the aortic diameter was measured with the operating microscope (Supplementary Material, Fig. S1). The primary endpoint was the ratio between the aortic diameter at AAA induction and at organ harvesting [14].

### Tenascin C immunohistochemistry

Formalin-fixed, paraffin-embedded samples harvested 3 weeks after AAA induction were sectioned at 2 µm thickness and stained according to instructions provided for the ABC staining kit (Vectastain Elite, Vector laboratories). Importantly, antigen retrieval was performed by immersing rehydrated sections in Tris-HCL buffer, pH 7.4, containing protease (Type XIV from *Streptomyces* *griseus*) at a concentration of 1.9 mg/ml at 37°C for 13 min. Incubation with primary antibody against TNC (ab19011, Abcam, 1:1000 dilution) was performed overnight at 4°C. Sections were imaged using an AxioImager.M2 microscope equipped with an AxioCam 512 colour camera (both Zeiss) at 10× magnification, and staining intensity was semi-quantitatively scored by an observer blinded to experimental conditions.

### Elastica van Gieson staining and analysis

Formalin-fixed, paraffin-embedded samples harvested 10 weeks after AAA induction were sectioned at 4 µm thickness and were stained according to the manufacturer’s (Morphisto GmbH, Frankfurt am Main, Germany) recommendations. The degree of elastin degradation was semi-quantitatively scored by 2 observers blinded to experimental conditions as previously described [15]. An average was calculated for each section.

### Human aortic vascular smooth muscle cell culture

Human aortic VSMC were cultured using M199 complete media supplemented with 20% foetal bovine serum and 1% Penicillin and Streptomycin. Cells were treated for 24 h under following conditions: (i) Control, (ii) 0.1 µM angiotensin II (Ang II) (Merck, Darmstadt, Germany), (iii) 10 µg/ml TNC (Merck, Darmstadt, Germany) and (iv) 10 µg/ml TNC in addition to 50 nM TAK-242 (Merck, Darmstadt, Germany). After the treatment, total RNA was isolated using RNeasy Mini kit (Qiagen, Hilden, Germany) and expression of target genes (related to AAA formation and linked to TNC [16], Supplementary Material, Table S1) were assessed by reverse transcription and quantitative polymerase chain reaction.

### Reverse transcription and quantitative polymerase chain reaction

Total RNA was transcribed into cDNA using QuantiTect reverse transcription kit (Qiagen, Hilden, Germany). Samples were measured in duplicates to a final reaction volume of 20 μl per well. The initial denaturation step of 5 min at 95°C was followed by 40 cycles of 15 s 95°C, 30 s 50°C and 30 s 72°C, using ROTOR-Gene Q (Qiagen, Hilden, Germany) and Rotor-Gene Q series software for computed tomography value analysis. GAPDH was used as housekeeping gene to normalize yielded Ct-values. Relative gene expression was calculated using 2−ΔΔCt method.

### Data acquisition and statistical analysis

All data are shown as mean ± standard deviation. One-way ANOVA with Tukey-HSD *post hoc* analysis as well as unpaired *t*-tests was used to compare means in different groups. Pearson correlation coefficient was calculated. *P*-values of <0.05 were considered significant. Levels of significance are shown as follows: *P < 0.05, **P < 0.01 and ***P < 0.001.

Data analysis and visualization were performed in R for Mac (R 3.0.2, The R foundation for Statistical Computing, Vienna, Austria). The following R packages were used: Hmisc, plotrix and splines.

## RESULTS

### Serum tenascin C levels in patients with abdominal aortic aneurysms

**Figure 1:**
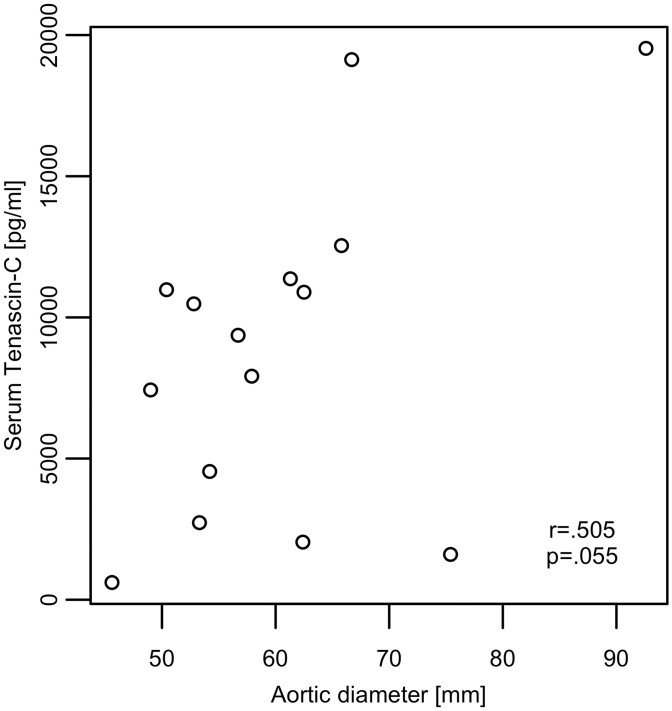
Correlation between aortic diameter (mm) and serum tenascin C levels (pg/ml) in patient cohort of AAA (*n* = 15).

**Figure 2:**
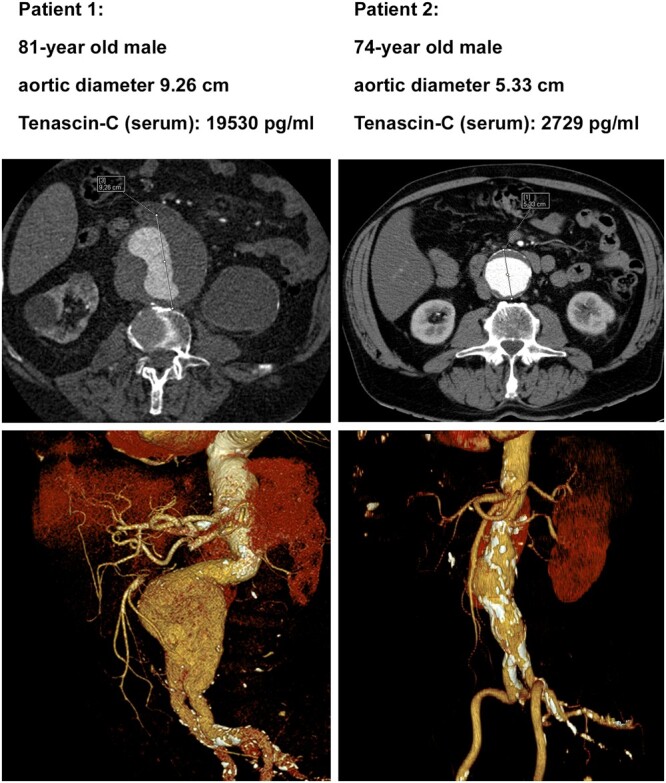
Representative three-dimensional reconstructed computed tomography images as well as tenascin C serum levels obtained from 2 abdominal aortic aneurysms patients.

A cohort of 18 patients with AAA was screened, of which 15 patients were included. Patient characteristics are shown in Table 1. The patients showed an average maximal aortic diameter of 60.4 ± 11.8 mm and average TNC levels of 8744 ± 5836 pg/ml. The serum level of TNC showed a trend of positive correlation with the maximal aortic dimension (Fig. 1; *r* = 0.505, *P* = 0.055). Representative computed tomography images and three-dimensional reconstructions with abdominal aorta dimension as well as serum TNC levels of 2 patients are shown in Fig. 2.

**Table 1:** Patient characteristics

Age (years)	67.0 ± 8.0
Gender (male)	15 (100%)
Body mass index	26.8 ± 2.8
Serum tenascin C (pg/ml)	8744 ± 5836
Maximum AAA diameter [mm]	60.4 ± 11.8
Aneurysm morphology	
Fusiform	12 (80%)
Saccular	3 (20%)
Positive family history	2 (13.3%)
Arterial hypertension	13 (86.7%)
Hyperlipidaemia	11 (73.3%)
Diabetes mellitus	2 (13.3%)
Peripheral artery disease	3 (20%)
Coronary artery disease	3 (20%)
Stroke	0
History of smoking	15 (100%)
Packyears	48.4 ± 29.6
COPD	6 (40%)

Data are presented as *n* (%) or mean plus standard deviation (*n* = 15).

AAA: abdominal aortic aneurysms; COPD: chronic obstructive pulmonary disease.

### Animal characteristics and aorta diameter in mice

**Figure 3:**
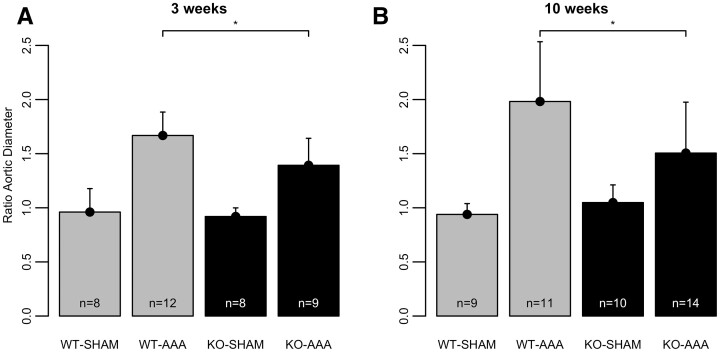
Aortic diameter ratio between the aortic size at AAA induction and during organ harvesting at 3 weeks (**A**) and 10 weeks (**B**) post-induction. Data are shown as mean plus standard deviation. *P < 0.05, for the level of significance in the comparison between WT-AAA and tenascin C KO-AAA (one-way ANOVA with Tukey-HSD *post hoc* test). AAA: abdominal aortic aneurysms; KO: knock out; WT: wild-type mice.

In total, 81 animals were used (WT-SHAM: *n* = 17, WT-AAA: *n* = 23, TNC KO-SHAM: *n* = 18, TNC KO-AAA: *n* = 23). No deaths due to aortic rupture or dissection were observed. The animals did not show any significant differences in body weight prior to AAA induction (WT-SHAM: 25.2 ± 1.8 g, WT-AAA: 24.8 ± 1.8 g, TNC KO-SHAM: 26.6 ± 3.1 g, TNC KO-AAA: 25.8 ± 2.5 g; n.s.). In WT and TNC KO mice, AAA groups showed a significant increase in aortic diameter ratio compared to sham-operated mice 3 and 10 weeks after AAA induction (3 weeks: WT-SHAM versus WT-AAA: *P* < 0.001, TNC KO-SHAM versus TNC KO-AAA: *P* < 0.05; 10 weeks: WT-SHAM versus WT-AAA: *P* < 0.001, TNC KO-SHAM versus TNC KO-AAA: *P* < 0.05, Fig. 3). Whereas no significant changes in diameter ratios were found in sham groups (3 weeks: WT-SHAM: 0.96 ± 0.22, TNC KO-SHAM: 0.92 ± 0.08, n.s.; 10 weeks: WT-SHAM: 0.94 ± 0.10, TNC KO-SHAM: 1.05 ± 0.16, n.s.), TNC KO mice with AAA showed a significantly lower diameter ratio compared to the WT group 3 weeks (TNC KO: 1.39 ± 0.25, WT: 1.67 ± 0.22, *P* < 0.05, Fig. 3A) and 10 weeks (TNC KO: 1.51 ± 0.47, WT: 1.98 ± 0.55, *P* < 0.05, Fig. 3B) after AAA induction, respectively.

### Tenascin C immunochemistry

**Figure 4:**
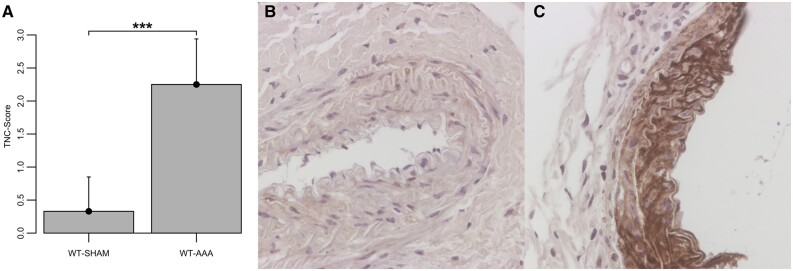
Expression of TNC 3 weeks after AAA induction (**A**) as well as representative images of TNC immunohistochemistry of the WT-SHAM (**B**) and the WT-AAA group (**C**). Data are shown as mean plus standard deviation. ***P < 0.001, for the level of significance in the comparison between WT-SHAM and WT-AAA (unpaired *T*-test). WT-SHAM: *n* = 6, WT-AAA: *n* = 6. Original magnification, 200×. AAA: abdominal aortic aneurysms; TNC: tenascin C; WT: wild-type mice.

TNC expression was markedly increased 3 weeks after AAA induction in WT mice compared to sham-operated mice (WT-SHAM: 0.33 ± 0.52, WT-AAA: 2.25 ± 0.69, *P* < 0.001, Fig. 4A). In WT mice with AAA TNC was mainly expressed in the tunica media. In addition, sham-operated WT mice and both sham-operated as well as AAA TNC KO mice did not show any specific TNC staining.

### Elastin structure

**Figure 5:**
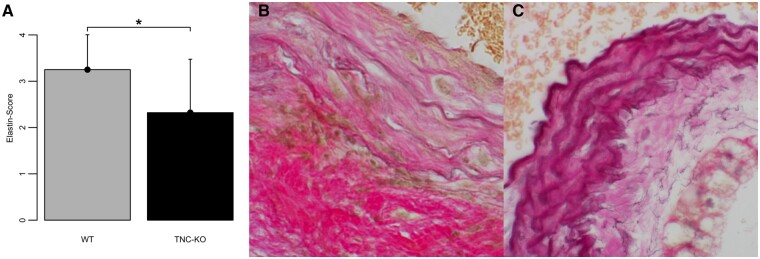
Scoring of elastin structure in Elastica van Gieson staining 10 weeks after AAA induction (**A**). Representative images of the WT-AAA group with severe elastin degradation and inflammatory cell infiltrates (**B**) and the TNC KO-AAA group with mild distention (**C**). Data are shown as mean plus standard deviation. *P < 0.05, for the level of significance in the comparison between WT-AAA and TNC KO-AAA (unpaired *T*-test). WT-AAA: *n* = 10, TNC KO-AAA: *n* = 14. Original magnification, 200×. AAA: abdominal aortic aneurysms; KO: knock out; TNC: tenascin C; WT: wild-type mice.

Sham-operated groups showed no signs of elastin degradation. AAA in WT mice had more degraded elastin fibres as well as focal infiltrates of leukocytes compared to TNC KO mice with AAA showing more dilated and less degraded elastin fibres (Fig. 5A–C; WT-AAA: 3.25 ± 0.75, TNC KO-AAA: 2.32 ± 1.15, *P* < 0.05).

### The effect of tenascin C on human aortic vascular smooth muscle cells

**Figure 6:**
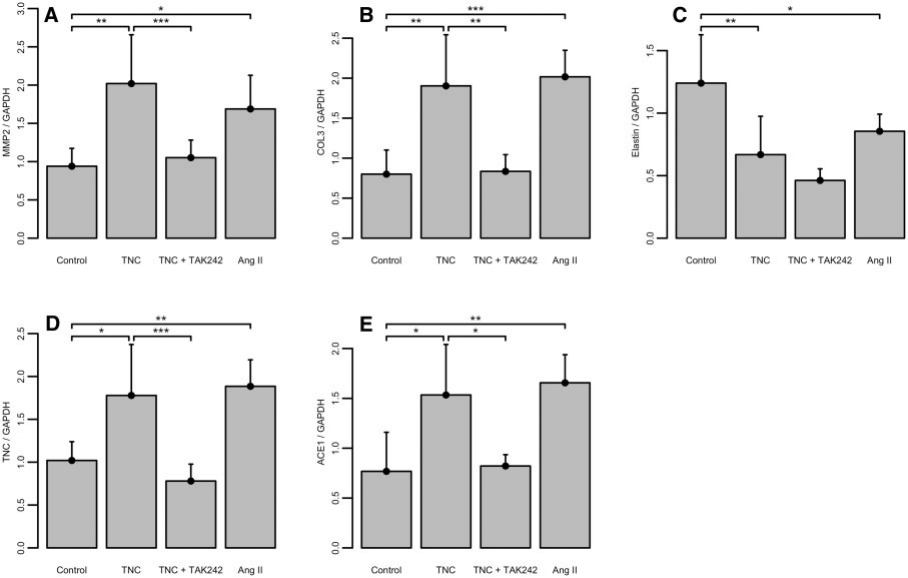
Expression matrix metalloproteinase 2 (**A**), Col3 (**B**), Elastin (**C**), TNC (**D**) as well as angiotensin-converting enzyme 1 (**E**) in human aortic vascular smooth muscle cells culture supernatant measured by reverse transcription and quantitative polymerase chain reaction after incubation with TNC. TNC+ TAK242 and Ang II. *P < 0.05, **P < 0.01 and ***P < 0.001, for the level of significance in the comparison between Control and TNC, TNC and TNC+TAK242 (1-way ANOVA with Tukey-HSD *post hoc* test) as well as Control and Ang II (unpaired *T*-test). TNC: tenascin C.

To further explain the role of TNC in AAA, human aortic VSMC were incubated with TNC or Ang II. Both conditions resulted in a massive up-regulation of matrix metalloproteinase 2 (Fig. 6A; Control: 0.94 ± 0.23, TNC: 2.02 ± 0.64, TNC+TAK242: 1.05 ± 0.23; Ang II: 1.69 ± 0.44; Control versus Ang II: *P* < 0.05, Control versus TNC: *P* < 0.01, TNC versus TNC+TAK242: *P* < 0.001) and COL3 (Fig. 6B; Control: 0.80 ± 0.30, TNC: 1.90 ± 0.64, TNC+TAK242: 0.84 ± 0.21; Ang II: 2.02 ± 0.33; Control versus Ang II: *P* < 0.001, Control versus TNC: *P* < 0.01, TNC versus TNC+TAK242: *P* < 0.01). More interestingly, administration of TNC as well as Ang II further increased the expression of TNC (Fig. 6D; Control: 1.02 ± 0.22, TNC: 1.78 ± 0.59, TNC+TAK242: 0.78 ± 0.20; Ang II: 1.89 ± 0.31; Control versus Ang II: *P* < 0.01, Control versus TNC: *P* < 0.05, TNC versus TNC+TAK242: *P* < 0.001) and angiotensin-converting enzyme 1 (Fig. 6E; Control: 0.77 ± 0.39, TNC: 1.53 ± 0.51, TNC+TAK242: 0.82 ± 0.11; Ang II: 1.66 ± 0.28; Control versus Ang II: *P* < 0.01, Control versus TNC: *P* < 0.05, TNC versus TNC+TAK242: *P* < 0.05) in VSMC. The effect of TNC depended on Toll-like receptor 4 (TLR-4) activation. Similar to that, the up-regulation of matrix metalloproteinase 2 and Col 3 by TNC was markedly declined in presence of the TLR-4 inhibitor TAK242. The expression of Elastin was significantly down-regulated after TNC and Ang II incubation. Hereby, the effect of TNC was not reversible by TLR-4 inhibition (Fig. 6C; Control: 1.24 ± 0.39, TNC: 0.67 ± 0.31, TNC+TAK242: 0.46 ± 0.09; Ang II: 0.86 ± 0.14; Control versus Ang II: *P* < 0.05, Control versus TNC: *P* < 0.01, TNC versus TNC+TAK242: n.s.).

## DISCUSSION

Selective and sensitive biomarkers for longitudinal monitoring of AAA disease activity are missing. Moreover, despite successful surgical and interventional repair, no causative pharmacological treatment for patients with AAA has been proven in randomized placebo-controlled trials. According to the results of our present study, we observed a consistent trend towards correlation between abdominal aortic diameter and TNC serum levels in patients with AAA. In line with this clinical finding in an experimental model of CaCl_2_-induced AAA, TNC deficient mice showed a significantly reduced AAA formation and progression as well as markedly reduced elastin disruption compared to WT mice. Mechanistically, TNC like Ang II induces the expression of ECM remodelling associated proteins as well as positive feedback loops in human aortic VSMC. This effect was partially reversed by TLR-4 inhibition.

TNC is a matricellular protein, which is expressed during embryogenesis, cancer and various remodelling processes. Its expression is modulated by cytokines, other matricellular proteins as well as mechanical stress [8]. VSMCs are the main source of TNC in the aorta. TNC expression is associated with the formation of AAA [3]. Furthermore, TNC was mainly expressed in the border zones of the AAA, mostly affected by inflammation and thereby may regulate the progression of AAA [3, 4, 17].

In the present study, we measured the levels of serum TNC in 15 patients with AAA. We could observe a trend towards a correlation between TNC levels and AAA diameter. Importantly, there are no evidence-based studies assessing serum TNC as prognostic marker for AAA. However, in patients with Type A dissection, we and others have demonstrated that TNC levels in plasma as well as in aortic tissue were elevated [5, 18, 19]. Furthermore, higher serum TNC levels were associated with increased in-hospital mortality after acute Type A and Type B dissection, suggesting the maladaptive role of TNC [20, 21]. These findings contradict another study, where elevated serum TNC levels on Day 7 after acute Type B dissection were associated with a lower risk for chronic aortic enlargement [22]. Further studies on larger populations need to elucidate the role of TNC as a marker for AAA progression as well as its time-dependent role in AAA and other aortic diseases.

Next, we wanted to test the hypothesis that TNC is involved in the progression of AAA. We used a model of CaCl_2_-induced AAA in WT and TNC-KO mice. In line with previous studies, TNC expression in AAA in WT mice was markedly localized in the border area and showed a massive up-regulation [3]. Previous investigations have already indicated that TNC was mostly expressed within mononuclear inflammatory cell infiltrates in human AAA associated with chronic inflammation and linked to neovascularization [23].

In our study, TNC deficient mice showed an attenuated AAA formation 3 and 10 weeks after periaortic CaCl_2_ application linked to a reduced elastin degradation. Our findings demonstrate contradictory results with the data of Kimura *et al.* [24]. Kimura *et al.* reported an increased rate of suprarenal dissections and ruptures in TNC KO mice in a model of CaCl_2_ induced AAA combined with Ang II infusions. The authors conclude that TNC acted protective on increased haemodynamic stress on the descending thoracic aorta due to CaCl_2_ induced abdominal aortic stiffening and Ang II infusions. Moreover, no significant differences between AAA diameter in TNC-KO and WT mice were found 6 weeks after CaCl_2_ induced AAA induction. Of importance, aortic size measurements were conducted *ex vivo* by histology and not compared to the baseline diameter in each animal [3]. It is important to stress that in the present study, we followed the animals for 3 and 10 weeks after CaCl_2_ induced AAA [24]. There was an increase in diameter over time and a significant difference between the aortic diameter in TNC KO animals as compared to WT after AAA induction.

In human aortic VSMC similarly to Ang II, incubation with TNC led to up-regulation of matrix metalloproteinase 2 and Col3 as well as to a down-regulation of Elastin. In combination with the increased Elastin degradation observed in WT mice with AAA, TNC could promote adverse aortic remodelling [25]. As a sign of positive feedback loop, Ang II as well as TNC induced TNC and angiotensin-converting enzyme 1 expression. Comparable effects of TNC could be observed in cardiac fibroblasts by our group [16]. These changes were partially reversed by the application of the TLR-4 antagonist TAK242. TNC is a known ligand of integrins as well as TLR-4 and subsequently interferes with matricellular proteins such as fibronectin facilitating cell migration [4, 26]. Additionally, it induces the NFκB signalling pathway via TLR-4 activation in various cell types including macrophages and fibroblasts, and consequently accelerates proinflammatory cytokine expression which also play a central role in AAA pathophysiology [2, 27, 28]. Based on these experimental results and the trend in our clinical cohort, we assume that TNC might play a maladaptive rather than beneficial role in the formation and progression of AAA.

### Limitations

Certain limitations need to be acknowledged. First, our patient cohort was small including only AAA patients, men only and therefore underpowered to detect a significant correlation between AAA diameter and TNC serum levels. This takes into account that men show an up to 3- to 6-fold increased prevalence of AAA [1, 29]. Moreover, only male animals were included. Second, CaCl_2_ induced AAA show aortic wall thickening and do not develop intraluminal thromboses compared to other models like the elastase model. However, the CaCl_2_ model does not require aortic clamping as well as an aortotomy and is therefore less invasive as well as less susceptible for induction flaws. Additionally, using the CaCl_2_ model, mice do not develop ruptures or aortic dissections. No further *in vivo* aortic size measurements (e.g. ultrasound) have been performed in addition to the intraoperative size evaluation [14, 30]. Third, we were not able to perform reverse transcription and quantitative polymerase chain reactions or other methods including MMP zymography on murine aortic samples because of the limited tissue. This limitation will be addressed in future studies.

## CONCLUSION

Higher serum TNC in patients with AAA might be associated with increased disease activity and aortic wall instability. Due to the preclinical observation with a trend of correlation between TNC expression and progression of AAA diameter, we suggest to prepare a larger clinical study to clarify the role of TNC as prognostic marker in AAA.

## SUPPLEMENTARY MATERIAL

Supplementary material is available at *ICVTS* online.

## Supplementary Material

ivac018_Supplementary_Data

## Abbreviations

**ABBREVIATIONS**
AAA: Abdominal aortic aneurysms
Ang II: Angiotensin II
CaCl_2_: Calcium chloride
KO: Knock out mice
TLR-4: Toll-like receptor 4
TNC: Tenascin C
VSMC: Vascular smooth muscle cells
WT: Wild-type mice
