# Supplementary material for: The expression and role of tenascin C in abdominal aortic aneurysm formation and progression
Source: Interact Cardiovasc Thorac Surg. 2022 Feb 8;34(5):841–8. doi: 10.1093/icvts/ivac018 (PMC9070497; doi:10.1093/icvts/ivac018)
Supplement: ivac018_Supplementary_Data [file ivac018_supplementary_data.zip › SupTab1.docx]

Supplementary Table 1 – List of primers sequences

| Gene | Forward 5’-3’ | Primer lenght (bp) | Reverse 5’-3’ | Primer lenght (bp) |
| --- | --- | --- | --- | --- |
| GAPDH | GGAAGGAAATGAATGGGCAG | 20 | CCCAATACGACCAAATCAGAG | 21 |
| MMP2 | AATGCCATCCCCGATAACC | 21 | TCCAAACTTCACGCTCTTCAG | 21 |
| Col3 | GGCTGAGTTTTATGACGGGC | 20 | GAGCGAGAAGTAGCCAGCTC | 20 |
| Elastin | GGGAAAGAGATGGGGGAAAG | 20 | GGAGGGGACAATTACGAAAG | 20 |
| TNC | AACGAACTGCCCATATCTCAG | 21 | TGGTTAGGTTTTCCAGAAGGG | 21 |
| ACE1 | CCCGGCAACTTTTCTGCTGAC | 21 | GATGTTGGTGTCGTGCGCC | 19 |

GAPDH: Glyceraldehyde 3-phosphate dehydrogenase, MMP2: Matrix metalloproteinase 2, Col3: collagen type III, Elastin: elastin, TNC: Tenascin C,
ACE1: Angiotensin-converting enzyme 1
