# Supplementary figures and images for: The expression and role of tenascin C in abdominal aortic aneurysm formation and progression
Source: Interact Cardiovasc Thorac Surg. 2022 Feb 8;34(5):841–8. doi: 10.1093/icvts/ivac018 (PMC9070497; doi:10.1093/icvts/ivac018)

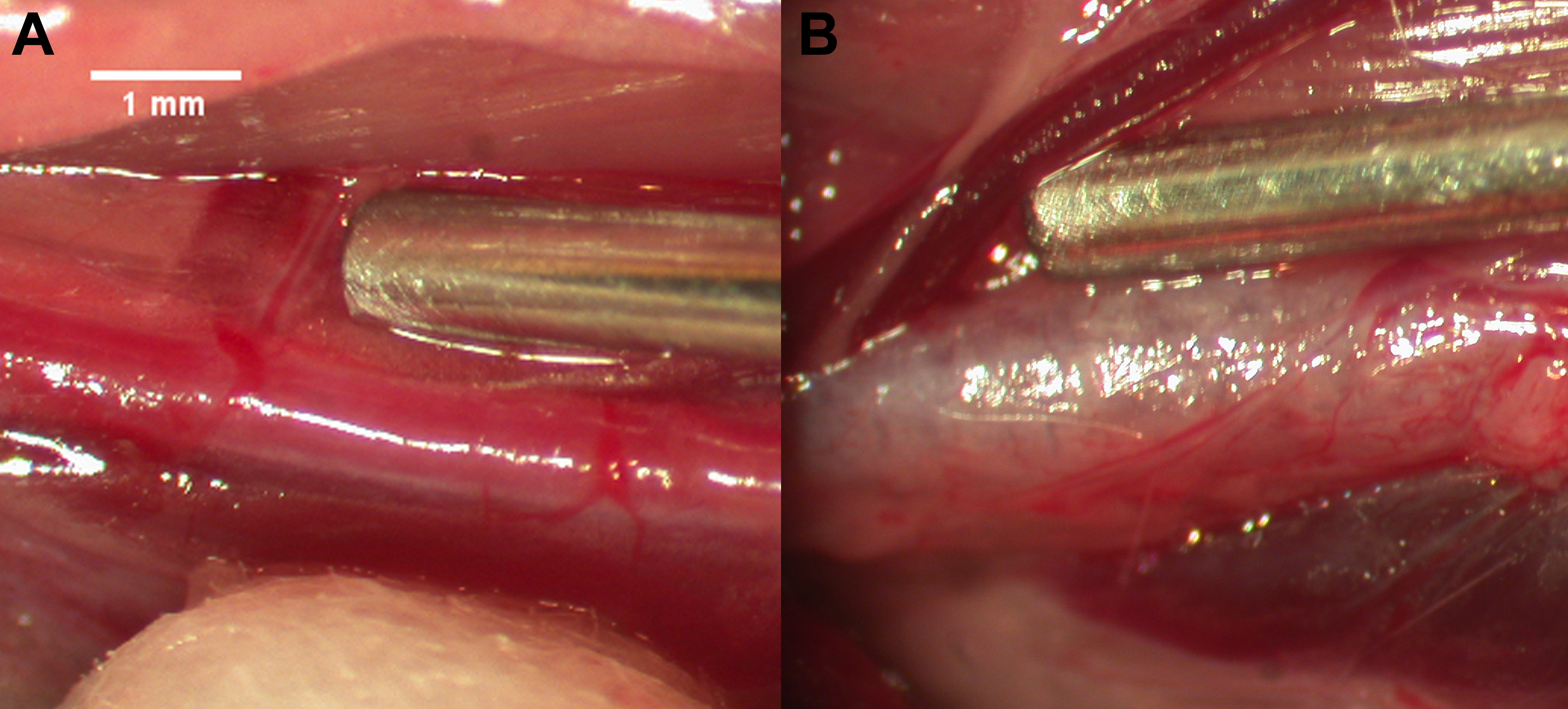

Supplement: ivac018_Supplementary_Data [file ivac018_supplementary_data.zip › SupFig1.tif]
